# Transcriptional Profiling the 150 kb Linear Megaplasmid of *Borrelia turicatae* Suggests a Role in Vector Colonization and Initiating Mammalian Infection

**DOI:** 10.1371/journal.pone.0147707

**Published:** 2016-02-04

**Authors:** Hannah K. Wilder, Sandra J. Raffel, Alan G. Barbour, Stephen F. Porcella, Daniel E. Sturdevant, Benjamin Vaisvil, Vinayak Kapatral, Daniel P. Schmitt, Tom G. Schwan, Job E. Lopez

**Affiliations:** 1 Department of Pediatrics, Section of Tropical Medicine, Baylor College of Medicine and Texas Children’s Hospital, Houston, Texas, United States of America; 2 Laboratory of Zoonotic Pathogens, Rocky Mountain Laboratories, National Institute of Allergy and Infectious Diseases, National Institutes of Health, Hamilton, Montana, United States of America; 3 Departments of Microbiology & Molecular Genetics, Medicine, and Ecology and Evolutionary Biology, University of California Irvine, Irvine, California, United States of America; 4 Genomics Unit, Research Technologies Section, Rocky Mountain Laboratories, National Institute of Allergy and Infectious Diseases, National Institutes of Health, Hamilton, Montana, United States of America; 5 Igenbio, Inc., Chicago, IL, United States of America; 6 Department of Molecular Virology and Microbiology, Baylor College of Medicine, Houston, Texas, United States of America; University of Kentucky College of Medicine, UNITED STATES

## Abstract

Adaptation is key for survival as vector-borne pathogens transmit between the arthropod and vertebrate, and temperature change is an environmental signal inducing alterations in gene expression of tick-borne spirochetes. While plasmids are often associated with adaptation, complex genomes of relapsing fever spirochetes have hindered progress in understanding the mechanisms of vector colonization and transmission. We utilized recent advances in genome sequencing to generate the most complete version of the *Borrelia turicatae* 150 kb linear megaplasmid (lp150). Additionally, a transcriptional analysis of open reading frames (ORFs) in lp150 was conducted and identified regions that were up-regulated during *in vitro* cultivation at tick-like growth temperatures (22°C), relative to bacteria grown at 35°C and infected murine blood. Evaluation of the 3’ end of lp150 identified a cluster of ORFs that code for putative surface lipoproteins. With a microbe’s surface proteome serving important roles in pathogenesis, we confirmed the ORFs expression *in vitro* and in the tick compared to spirochetes infecting murine blood. Transcriptional evaluation of lp150 indicates the plasmid likely has essential roles in vector colonization and/or initiating mammalian infection. These results also provide a much needed transcriptional framework to delineate the molecular mechanisms utilized by relapsing fever spirochetes during their enzootic cycle.

## Introduction

The causative agents of Lyme disease and relapsing fever borreliosis are examples of pathogens that have adapted divergently in ixodid and argasid tick vectors, respectively. Lyme disease associated species such as *Borrelia burgdorferi*, *Borrelia garinii*, and *Borrelia afzelii* infect *Ixodes* ticks, while most Ethiopian (*Borrelia duttonii*, *Borrelia crocidurae*), Palearctic (*Borrelia hispanica*) and Nearctic (*Borrelia hermsii*, *Borrelia parkeri*, and *Borrelia turicatae*) relapsing fever spirochetes are transmitted by *Ornithodoros* species. The life cycle and feeding behavior between *Ixodes* and *Ornithodoros* ticks differ significantly, and consequently each pathogen has evolved to colonize specific tick vectors. *B*. *burgdorferi* persistently colonizes the midgut after ticks ingest spirochetes [1, 2]. During the subsequent bloodmeal, the pathogens exit the midgut and transiently migrate through the salivary glands and infect mice after 36 hours of tick attachment [1–5]. As *B*. *burgdorferi* transits through the tick to the mammal, unique patterns of plasmid gene expression are associated with spirochete adaptation within different host environments [6–12].

Relapsing fever spirochetes enter the midgut of *Ornithodoros* species during an acquisition bloodmeal and a population subsequently disseminates to the salivary glands within 10–14 days [13]. During the following bloodmeal, the spirochetes are quickly transmitted as ticks attach [14, 15], suggesting that genes expressed by the bacteria in the salivary glands may have a role in colonizing the tissues and/or initiating early mammalian infection [16]. However, differential expression that occurs within the tick and mammal remain largely unknown because of small but complex spirochete genomes.

Relapsing fever spirochetes are comprised of a linear chromosome, a 150 to 174 kb megaplasmid and 6–16 linear and circular plasmids that are ~10–55 kb [17–20]. The plasmids contain extensive regions of repetitive DNA, which has resulted in incomplete assemblies [21, 22]. *B*. *turicatae* was first sequenced by Sanger based methods and the chromosome and a 114 kb of the ~150 kb linear megaplasmid (lp150) [23] were deposited into the National Center for Biotechnology Information (NCBI). From this assembly, custom Affymetrix GeneChips were developed. However, the genome remained in 118 contigs [24] and the 3’ end of lp150 was still unassembled.

While the ~10–55 kb linear plasmids contain genes that are temperature regulated and code for surface proteins involved with antigenic variation [17, 20, 25, 26], less understood is the megaplasmid’s role in relapsing fever spirochete pathogenesis. In particular, differential expression of open reading frames (ORFs) during the tick-mammalian infectious cycle remains unclear. In this study, genomic and transcriptomic shortcomings for relapsing fever spirochetes were addressed to further assemble *B*. *turicatae* lp150 and identify ORFs up-regulated during vector colonization. New sequencing technology generated long scaffolds and resulted in the most complete version of lp150. A microarray analysis of megaplasmid’s ORFs was also performed to identify transcriptional changes occurring in the tick and mammal. Gene expression profiles were first compared between *B*. *turicatae* grown at 22°C (a temperature mimicking the tick) to the spirochetes cultured at 35°C and bacteria isolated from murine blood. This approach circumvented difficulties associated with low recovery of spirochete RNA from the tick vector (unpublished findings). *In vitro* transcriptional analyses were validated in the tick and mammal of ORFs within a locus at the 3’ end of lp150 that were up-regulated at 22°C and predicted to code for surface proteins. This comprehensive transcriptional analysis of lp150, provides supportive evidence for the importance of this plasmid in vector colonization and preadapting the pathogens for entry into the mammal. Importantly, gene candidates that encode for putative surface proteins have been identified to functionally characterize their contribution in vector colonization and transmission.

## Methods and Materials

### Ethics Statement

Murine studies were approved by the Institutional Animal Care and Use Committee (IACUC) at Baylor College of Medicine, protocol number AN-6563 and AN-6580, within the Association for Assessment and Accreditation of Laboratory Animal Care and the National Institutes of Health Office of Laboratory Animal Welfare assured program. Animal husbandry was provided by veterinary staff and technicians. Mice were housed in BSL-2 approved facilities on a 12 hour light cycle, and checked daily for food and water. While protocols had humane endpoints if mice display immobility, huddled position ruffled fur, self-mutilation, and notable weight loss, animals did not become severely ill or were euthanized prior to the end of experiments.

### Spirochete strains, pulse-field electrophoresis, and southern blotting

Isolation of genomic DNA, pulse-field electrophoresis, and southern blotting were performed as previously described [19, 27, 28]. The origins of *B*. *turicatae* strains 91E135 (Oz1), 95PE-570, 99PE1807, TCB-1, and FCB were described previously [19]. Genomic DNA was separated on an agarose gel and transferred to MagnaGraph Nylon Transfer Membrane (Osmonics Inc., Minnetonka, MN USA). For southern blots, a hybridization probe was produced using the PCR DIG synthesis kit (Roche Applied Science, Indianapolis, IN, USA) to *bta128*, an ORF on a 28.0 kb contig that was suspected to localize to the 3’ end of lp150.

### DNA sequencing and annotation

The partially assembled version lp150 that was generated from Sanger shotgun sequencing [24] in the Rocky Mountain Laboratories Genomics Unit led to resequencing the *B*. *turicatae* genome using a Pacific Biosciences RS I Single Molecule, Real-Time (PacBio SMRT) DNA approach (Pacific Biosciences, Menlo Park, CA, USA). Genomic DNA was purified with Genomic Tip 500/G columns (Qiagen) and 7 μg was sheared using the g-Tube (Covaris, Woburn, MA, USA), following the PacBio protocol for low-input (10 kb) preparation and sequencing. AMPure magnetic beads were used to remove salt according to the PacBio template and preparation sequencing instructions. SMRTbell Template Preparation kit (Pacific Biosciences) was used to construct a 3–10 kb library and the size determined using a 2100 Bioanalyzer (Agilent). The Pacific Biosciences calculator (version 2.0.1.2) was used to determine the amount of primer and polymerase (DNA/Polymerase binding kit 2.0), and samples were sequenced with the MagBead Seq v1 protocol (Pacific Biosciences). Sequence reads were assembled using the PacBio SMRT Analysis software (version 2.1.1), and default filters removed reads < 50 bases and < 0.75 accuracy.

*De novo* genome assembly was accomplished in four successive steps using the RS_HGAP_assembly.1 protocol. Contigs were aligned against three other reiterative assemblies that were generated by Sanger shotgun sequencing using the Genome Align tool, which is based on basic local alignment search tool (BLASTN) in ERGO^™^ (IgenBio, Inc, Chicago, IL, USA), and annotated using the ERGO^™^ Annotation Pipeline [29, 30]. A 30 amino acid cutoff was used to predict ORFs, and lp150 was deposited to the public NCBI database (HM008710.3).

### Analysis and annotation of lp150 sequence

The ERGO^™^ Bioinformatics Suite was used to search for direct and inverted repeats (Igenbio, Inc.), while the Vector NTI Advance 11 (Life Technologies) program performed amino acid alignments of the proteins encoded in the 3’ end of lp150. Paralogous gene families were determined by pairwise protein alignments and defined as at least 60% amino acid identity [31]. BLAST, PROSITE database, TMHMM 2.0, and SignalP 4.1 were used to identify putative homologues, amino acid domains, transmembrane regions, and signal peptides, respectively. To determine the presence of putative homologues between species of relapsing fever spirochete in which the megaplamids had been assembled, BLAST analysis was performed with ORFs localized on the 3’ end of lp150.

### Infecting mice and *O*. *turicata* with *B*. *turicatae*

Animal studies, *O*. *turicata* rearing conditions, and infecting cohorts of ticks were performed as previously detailed [32]. A mouse was initially needle inoculated intraperitoneally with 1 x 10^5^ *B*. *turicatae* and monitored daily. Upon spirochete visualization in the blood by dark field microscopy, the animal was sedated by intraperitoneal injection with 25 mg/ml Ketamine and 7.6 gm/ml Rompun at a dosage of 0.1 ml per 25 gm of body weight, and a cohort of uninfected second stage nymphs were allowed to engorge [32]. After ticks had molted into a third nymphal stage, a cohort of 10 was used to infect mice by allowing them to feed to repletion. Blood was inspected daily for the presence of spirochetes by dark field microscopy, and upon visualization of *B*. *turicatae*, animals were sedated, exsanguinated by cardiac puncture, 2.5 μl collected for spirochete quantification [32], and the blood processed for isolation of spirochetes and RNA.

### Sample processing, RNA isolation, and amplification

RNA was isolated from *B*. *turicatae* 91E135 cultured at 22°C and 35°C, infected and uninfected unfed ticks, and murine blood five days after tick bite, as previously described [32]. Spirochete cultures (500 ml, passaged 10 times after their initial isolation) were grown at 22°C and 35°C in Barbour-Stoenner-Kelly (BSK) medium containing 12% rabbit serum [33]. When spirochetes attained 1 x 10^7^ bacteria per ml, the cultures were centrifuged for 15 min at 10,000 x g, the supernatant removed, and the bacterial pellet resuspended and incubated for 5 min with 100 ml RNAprotect Cell Reagent (Qiagen Inc., Valencia, CA, USA). Spirochetes were subsequently centrifuged at 6,000 x g for 15 min, the supernatant removed, and pellets frozen in a dry ice and ethanol bath and stored at -80°C until RNA extraction. During centrifugations, temperatures were maintained at 22°C or 35°C depending on the previous growth conditions.

To concentrate *B*. *turicatae* from murine blood, animals were exsanguinated as stated above, and whole blood centrifuged at 5,000 x g for 10 min at 35°C. The plasma containing *B*. *turicatae* was removed and placed in a RNAase-free centrifuge tube. Serum was centrifuged at 15,000 x g for 10 min at 35°C, the supernatant removed and discarded, the spirochete-enriched pellet was resuspended in 100 μl of RNAlater RNA Stabilizing Reagent (Qiagen), and flash frozen with liquid nitrogen. Spirochetes were kept at -80°C until RNA was isolated.

After ticks acquired *B*. *turicatae* upon molted, total RNA was isolated from three cohorts of five third stage nymphal *O*. *turicata* as previously described [32]. The ticks were placed into RNAse free centrifuge tubes (Qiagen) and flash frozen in liquid nitrogen, triturated into a powder with an RNAse free pestle, and suspended in 100 μl of RNAlater RNA Stabilization Reagent (Qiagen).

RNA was extracted using the RNeasy Mini kit (Qiagen) following the manufacturer’s instructions. RNA integrity numbers (RIN) were calculated with an Agilent RNA 6000 Pico kit and the 2100 Bioanalyzer (Agilent Technologies, Waldbroon, Germany). One hundred ng of input RNA with RIN values above 8.0 was used for a nonbiased amplification with Ambion’s MessageAmp^™^ II- Bacteria Prokaryotic RNA kit (Life Technologies, Foster City, CA, USA) following the manufacturer’s instructions. *In vitro* transcription was performed to incorporate biotin-11-CTP (PerkinElmer Life Sciences, Waltham, MA, USA) into the cDNA, and 3 μg of the sample was subsequently fragmented using Ambion’s 10x Fragmentation Reagent (Life Technologies) and used for each Affymetrix GeneChip (Affymetrix, Santa Clara, CA, USA).

### Microarrays and data analysis

Custom Affymetrix GeneChips were developed for the Rocky Mountain Laboratories (RML) using *B*. *turicatae* probe sets representing 1,387 ORFs. GeneChips were generated from the partially assembled Sanger shot-gun sequencing effort [24]. Amplified and labeled cDNA was used to hybridize to the GeneChips, and three biological replicates per condition were performed according to manufacturer’s instructions. Expression Console (www.affymetrix.com) was used for normalization along with GeneSpring (www.chem.agilent.com) and Partek Genomics Suite (Partek Inc., St. Louis, MO, USA) for quality evaluation of replicates and condition grouping. The data were imported into Partek Genomics Suite software (Partek Inc., St. Louis, MO, USA) to produce a principal components analysis (PCA) plot to further confirm grouping of biological replicates. An ANOVA was performed within Partek Genomics Suite to obtain multiple test-corrected p-values using the false discovery rate method [34] at a 0.05 significance level, and was combined with fold change to determine the genes that were differentially expressed. Expression data were submitted to the Gene Expression Omnibus in NCBI (GSE69041).

### Primer and probe design, and quantitative reverse transcriptase PCR (qRT-PCR)

Differential expression of a gene cluster on the 3’ end of lp150 at 22°C versus 35°C, and in the tick compared to spirochetes isolated from murine blood was performed by qRT-PCR as previously detailed [32]. Primers and probe sets (TIB MOLBIO, LLC, Adelphia, NJ, USA) were designed specifically to all but three genes, *bta117* to *bta119* (S5 Table), and were used at 200 μM and 150 μM concentrations, respectively. Similar genomic equivalents between *flaB*, the gene used for normalization, and *bta* genes were determined by performing qRT-PCR assays with *B*. *turicatae* 91E135 DNA at an input of 0.3, 3, and 30 ng [32]. Additionally, RNA purity was evaluated for DNA contamination by performing assays without RT enzyme. qRT-PCR assays using RNA from spirochetes cultivated at 22°C and 35°C were performed in triplicate with 0.3, 3, and 30 ng, while 150 ng of RNA was used from infected ticks and blood. The 2^-*ΔΔCT*^ equation ascertained a > 2-fold change in gene expression [32]. Ninety-five percent confidence intervals for each ORF were established under a given condition, and the subsequent fold-change determined. To evaluate statistical significance in expression between conditions (22°C versus 35°C and infected ticks versus blood), replicate data were linearized by the 2^-CT^ equation, means and standard deviation determined, and a Student’s *t* test performed as previously described [35].

## Results

### Assembly of the large linear megaplasmid

**Fig 1:**
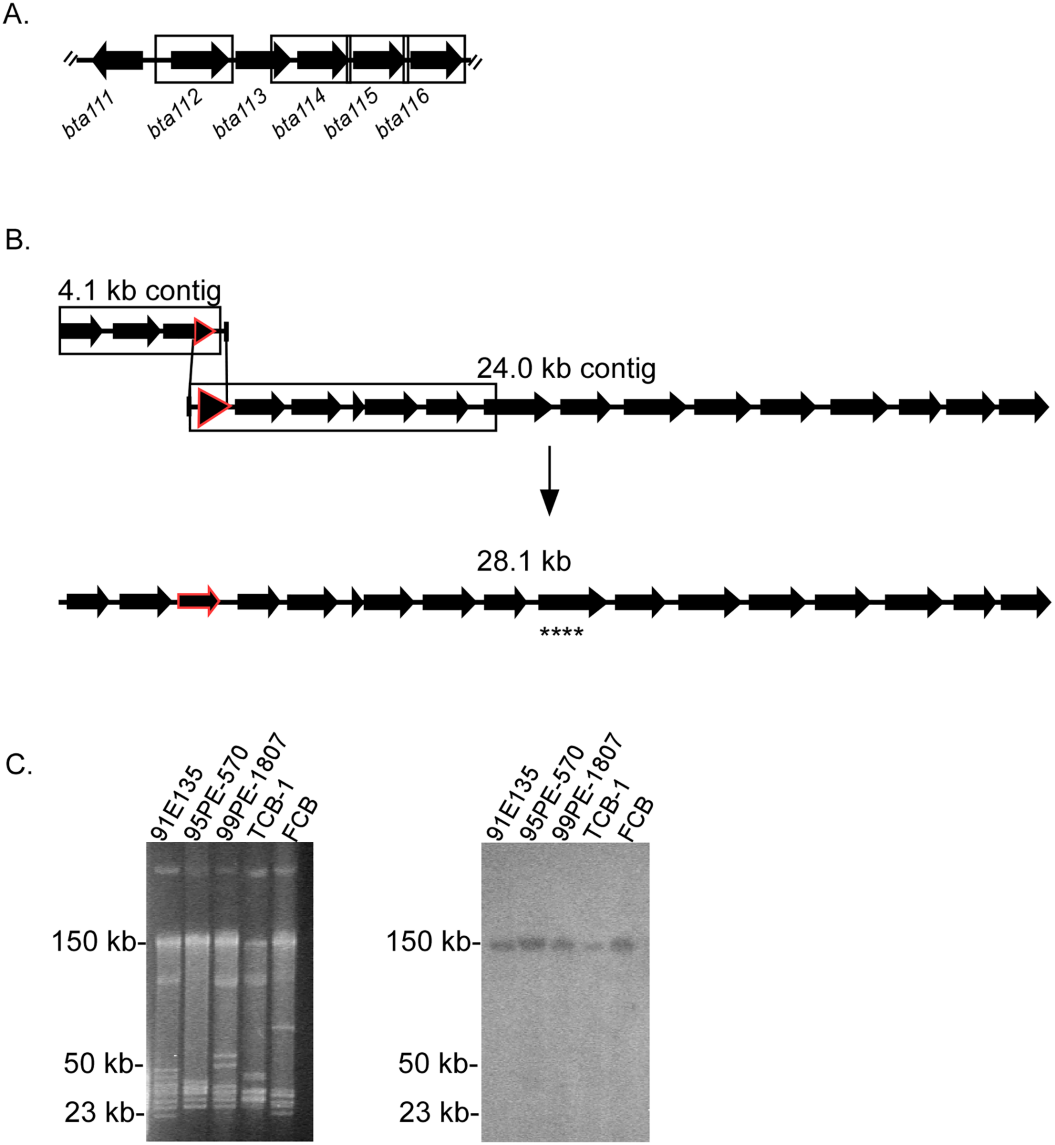
Identification and assembly of two contigs that comprise the 3’ end of lp150. Six genes at the right end of the partially assembled lp150 (*bta111* to *bta116*) are shown as arrows, while boxes represent regions of direct and inverted repeats that share similarity between a 4.1 and 24.0 kb contig (A and B). The red highlighted arrows on the 4.1 and 24.0 kb contigs represent nucleotide identity that led to the assembly of a 28.1 kb contig (B). The location of a southern blot probe designed for the 28.1 kb contig is represented by four asterisks (B). A pulse-field agarose gel (C, left) and southern blot (C, right) display the genetic profile and localization of the 28.1 kb contig to lp150, respectively. *B*. *turicatae* isolates are shown on the top (C), and molecular size standards are on the left of each image.

Close sequence analysis of the 118 contigs from the partially assembled *B*. *turicatae* genome indicated that one of the contings belonged to the 3’ end of the 150 kb plasmid. Over 50% amino acid identity was observed between Bta116 (the protein encoded on the far right end of the partially assembled lp150) and ORFs on the 4.1 and 24.0 kb contigs (Fig 1A and 1B). The ends of the 4.1 and 24.0 kb contigs shared 336 nucleotides and were manually assembled into a 28.1 kb contig (Fig 1B). Similar nucleotide repeats were detected throughout the 3’ end of the 114 kb partial plasmid and within the 28.1 kb contig (Fig 1A and 1B). This suggested that given the regions of nucleotide similarity and the cumulative molecular size of the two contigs, the 28.1 kb contig may localize to the 3’ end of the partially assembled lp150. Southern blotting with a probe designed within the 28.1 kb contig confirmed its localization to the 150 kb linear megaplasmid, and indicated that the region is conserved among the tested isolates (Fig 1B and 1C). PCR using primers designed to span the gap between the partially assembled lp150 and 28.1 contig was unsuccessful (data not shown), thus we sequenced the genome utilizing PacBio SMRT technology.

**Fig 2:**
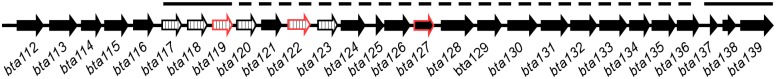
Assembly of ORFs (*bta117* to *bta139*), shown as arrows, toward the 3’ end of lp150. Paralogues are represented with a vertical lines (b*ta117* to *bta120*, *bta122*, and *bta123*) or red outline (*bta119*, *bta122*, and *bta127*). Solid horizontal lines are above ORFs (*bta117* to *bta119*) that spanned the gap between the partially assembled plasmid and 28.1 kb contig (dotted line), and those that were assembled at the end of the plasmid (*bta137* to *bta139*).

The total number of reads generated by the PacBio SMRT approach was 11,373 with an average sequence read of 3,230 bp (S1 Fig), and a 33.5 X coverage. Aligning the contigs to those generated by Sanger-based methods [24], condensed the genome from 118 to 24 contigs (data not shown). In total, 148,577 bp of the ~150 kb plasmid were accounted for. Sixteen ORFs were reannotated from the previously published version of lp150 [23], and categorized as A, B, or C following their *bta* numerical designation (S1 Table). Twenty-three ORFs, most of which were within the 28.1 kb contig, were added to the 3’ end of the plasmid. Nomenclature was kept consistent to that described by Miller et al. [23], and the 23 ORFs were designated *bta117* to *bta139* (Fig 2). In total, 155 ORFs were localized to lp150 (S1 Table).

### *In silico* evaluation of the 3’ end of lp150

**Fig 3:**
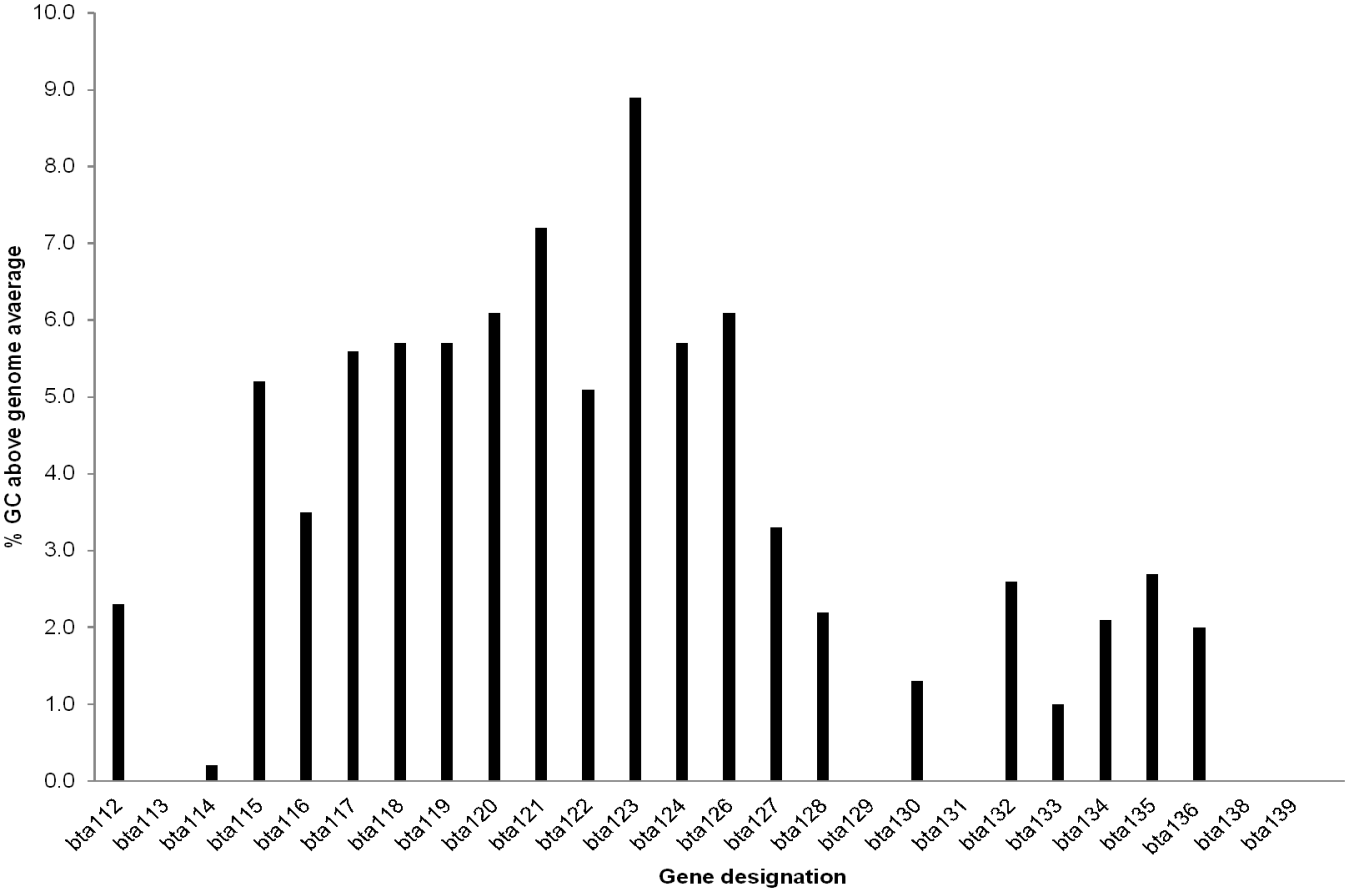
Percent increase of GC content of *bta112* to *bta139* compared to the *B*. *turicatae* genome. *bta125* and *bta137* were excluded from the analysis given the genes may be pseudogenes.

With ORFs on the partially assembled lp150 previously annotated and evaluated between several species of relapsing fever spirochete [23], efforts were focused to further assess the 3’ end of the plasmid. Pairwise comparisons identified paralogues in the 3’ end of lp150. Bta117 to Bta120, Bta122, Bta123, and Bta127 were paralogues [31], given a 63.5 to 82.2% amino acid identity (Fig 2 and S2 Table). Furthermore, assessment of *bta112* to *bta139* indicated that 18 ORFs had 2 to 9% increases in GC content over the genome average of 29.5% (Fig 3). Patterns were also identified where the genes were flanked by two, three to seven nucleotide direct repeats, and 21 to 29 nucleotide inverted repeats (S3 Table).

BLAST analysis indicated that apart from *B*. *parkeri*, a closely related North America species [19, 21], homologues with > 42% amino acid identity were absent for most proteins encoded toward the 3’ end of lp150 (Table 1). Moreover, putative functional homologues were not identified for the proteins. SignalP 4.1 and the Prosite database predicted signal peptides or lipoprotein motifs for 23 of 28 proteins of the lp150 3’ end (Table 1). Bta115 and Bta121 were the only two proteins with conserved domains (Table 1), a spectrin (SPEC) repeat (E-value of 10^−3^) and a procyclic acidic repetitive protein (PARP) domain (E-value of 10^−7^), respectively.

**Table 1:** Percent amino acid identity between *B*. *turicatae* Bta proteins located on the 3’ end of lp150 to *B*. *parkeri*, *B*. *hermsii* and *B*. *duttonii*.

	*B*. *parkeri*	*B*. *hermsii*	*B*. *duttonii*	Signal peptide or lipoprotein motif	Predicted domain
Bta112	72	34	24	+	-^A^
Bta113	89	31	25	+	-^A^
Bta114	80	33	-^A^	+	-
Bta115	71	28	-	+	SPEC superfamily
Bta116	86	26	-	+	-
Bta117	72	-	-	+	-
Bta118	74	27	-	+	-
Bta119	73	27	27	+	-
Bta120	82	26	33	+	-
Bta121	60	-	-	+	TRYPAN PAR
Bta122	73	25	25	+	-
Bta123	69	27	-	+	-
Bta124	55	25	-	+	-
Bta125	50	24	-	-	-
Bta126	72	27	23	+	-
Bta127	35	32	24	+	-
Bta128	37	33	28	+	-
Bta129	32	34	25	-	-
Bta130	34	34	26	-	-
Bta131	24	37	22	+	-
Bta132	38	42	27	+	-
Bta133	31	33	27	+	-
Bta134	29	30	26	+	-
Bta135	36	31	26	+	-
Bta136	34	34	26	+	-
Bta137	-	-	-	-	-
Bta138	-	45	-	-	-
Bta139	37	35	29	+	-

^A^. -, homologue, motif, or predicted domain not detected

### Microarray analysis of ORFs on lp150

Microarray GeneChips were originally developed using the partially assembled genome of *B*. *turicatae*, and consequently probes for lp150 were designed for 112 of 155 ORFs. Comparing transcriptional profiles of *B*. *turicatae* grown at 35°C to spirochetes isolated from murine blood indicated that 62.5% (70/112) of ORFs in which probes were designed to lp150 were similarly expressed in both conditions (S2 Fig and S4 Table). Also, 30.4% (34 of 112) of ORFs were uniquely up-regulated > 2-fold at 35°C relative to infected blood, while we detected an increase of expression > 2-fold for 7.1% (8 of 112) in the blood compared to 35°C (S2 Fig and S4 Table).

To investigate possible adaptations of *B*. *turicatae* to its tick vector, we assessed transcriptional profiles of spirochetes grown *in vitro* at 22°C (representing unfed tick conditions) to those either cultivated at 35°C (to mimic the temperature of mammalian blood) or bacteria isolated from murine blood. Distinct gene expression was observed for *B*. *turicatae* grown at 22°C compared to spirochetes cultivated at 35°C, with 25.8% (29/112) of ORFs similarly expressed under both conditions, and 74.1% (83/112) up-regulated > 2-fold at 22°C relative to 35°C (S2 Fig and S4 Table). We did not detect an ORF with a statistically significant value to be up-regulated at 35°C compared to spirochetes grown *in vitro* at 22°C (S2 Fig and S4 Table). These results indicate that a majority of ORFs on lp150 are up-regulated at a temperature similar to the tick environment.

**Fig 4:**
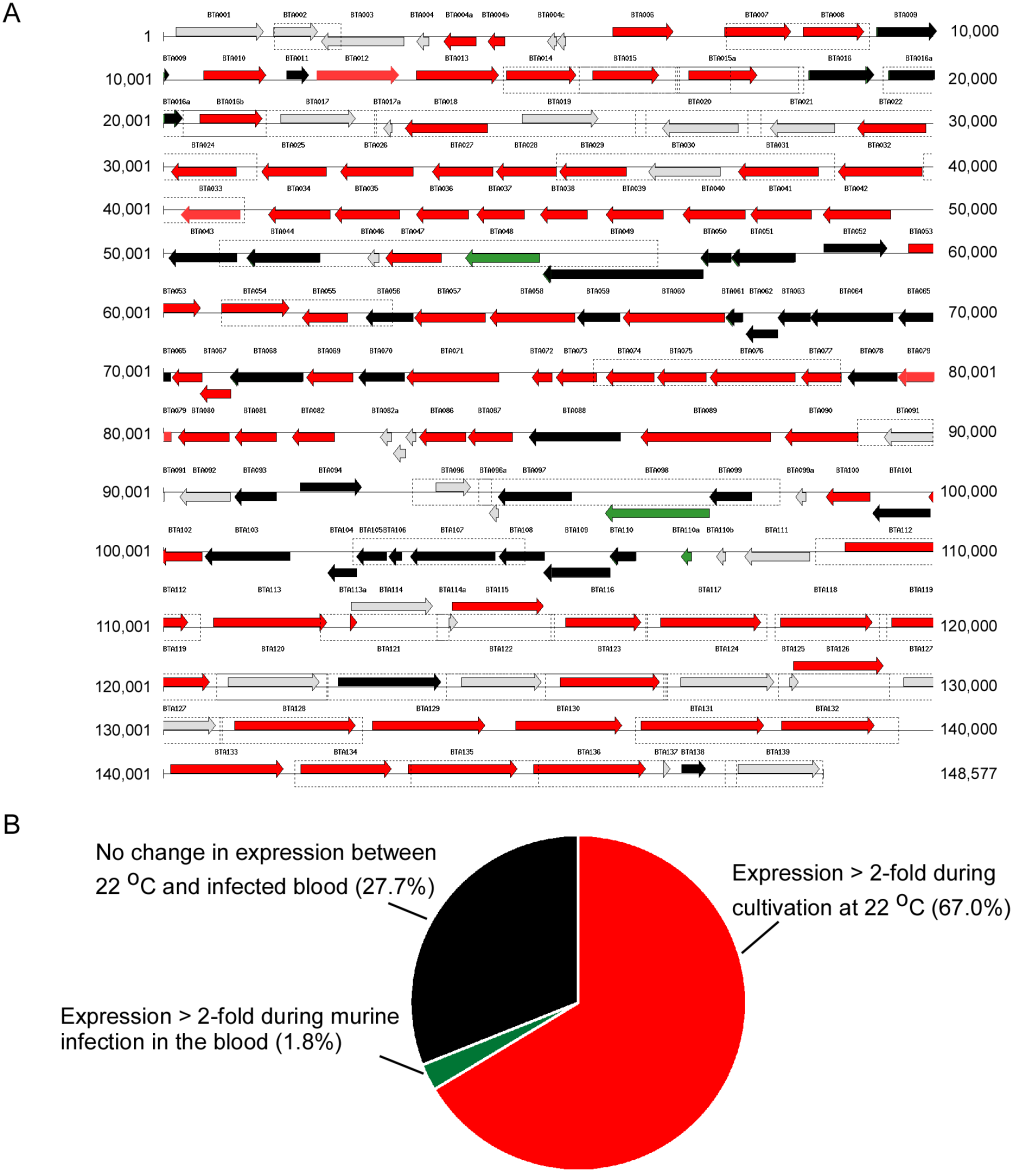
Transcriptional profile of lp150. Grey shaded arrows indicate ORFs that were not present on microarrays, while red and green indicate ORFs that were up-regulated at 22°C and the blood, respectively (A). Black arrows represent ORFs that were not differentially expressed under the two conditions or the transcript signal was below negative controls (A). The nucleotide number is displayed on the right and left of the image, while ORF designations are shown above the arrows. Also shown are the ratios of ORFs on lp150 that are up-regulated in the blood, 22°C, and those not differentially expressed (B).

Transcriptome analysis of *B*. *turicatae* cultivated at 22°C compared to spirochetes isolated from murine blood indicated that 67% of the ORFs were up-regulated by the spirochetes during *in vitro* growth conditions mimicking the tick environment (Fig 4A and 4B). Of these, three loci were identified on lp150 that contained clusters of seven or more ORFs (Fig 4A and 4B and S4 Table) that were up-regulated: *bta022* to *bta042*, *bta071* to *bta077*, and clusters of genes from *bta112* toward the end of the plasmid (Fig 4A). Also, *bta100* was the only ORF up-regulated at 22°C with a provisionally annotated function: a putative ATPase involved in plasmid partitioning. Three ORFs (*bta048*, *bta098*, and *bta110a*) were up-regulated > 2-fold by *B*. *turicatae* that was isolated from the blood compared to spirochetes cultivated at 22°C (Fig 4A and 4B). *bta048* was previously annotated as the beta subunit of the ribonucleoside-diphosphate reductase, while *bta098* contained a phage terminase-like family Pfam domain (PF03237) [23]. The remaining ORF, *bta110a*, coded for a protein of unknown function.

### *In vitro* and *in vivo* assessment of lp150 ORF subsets by qRT-PCR

While we were unable to isolate sufficient amounts of RNA from infected *O*. *turicata* for microarrays (data not shown), expression analysis of individual ORFs in the tick by qRT-PCR was possible. ORFs toward the 3’ end of lp150 (*bta112 to bta139*) were further evaluated in the tick given that most were predicted surface proteins, which suggest functional significance. Of these, six ORFs (*bta114*, *bta120*, *bta122*, *bta124*, *bta127*, and *bta139*) were absent from the custom microarrays and their expression was investigated. Bta121 was also of interest because it contained a PARP domain similar to the *Trypanosoma* procyclin that is highly expressed by parasites in their vector [36, 37]. However, microarrays suggested that *bta121* expression was not temperature dependent (S4 Table), and this finding led us to further evaluate the gene.

For qRT-PCR assays, given that *bta125* and *bta137* coded for 40 and 30 amino acid proteins, respectively, their size suggested that they may be silent ORFs and were omitted from the remaining analyses. Primer and probe sets (S5 Table) were optimally designed specifically to the remaining target ORFs except for *bta117* to *bta119* and *bta139*. With 76.2 to 81.3% nucleotide identity and repeats within *bta117* to *bta119*, we were unable to identify unique regions from each gene. *flaB* was used as the gene for normalization based on previous findings demonstrating similar expression between spirochetes grown at 22°C and 35°C (S3 Fig) [32, 38]. Similar genomic equivalents between *flaB* and the target ORFs were confirmed by performing qPCR with genomic DNA template (S4 Fig), which also indicated that primers and probe sets were specific to a given ORF. RNA preparations were confirmed to be free of contaminating DNA by performing assays lacking reverse transcriptase (data not shown).

**Fig 5:**
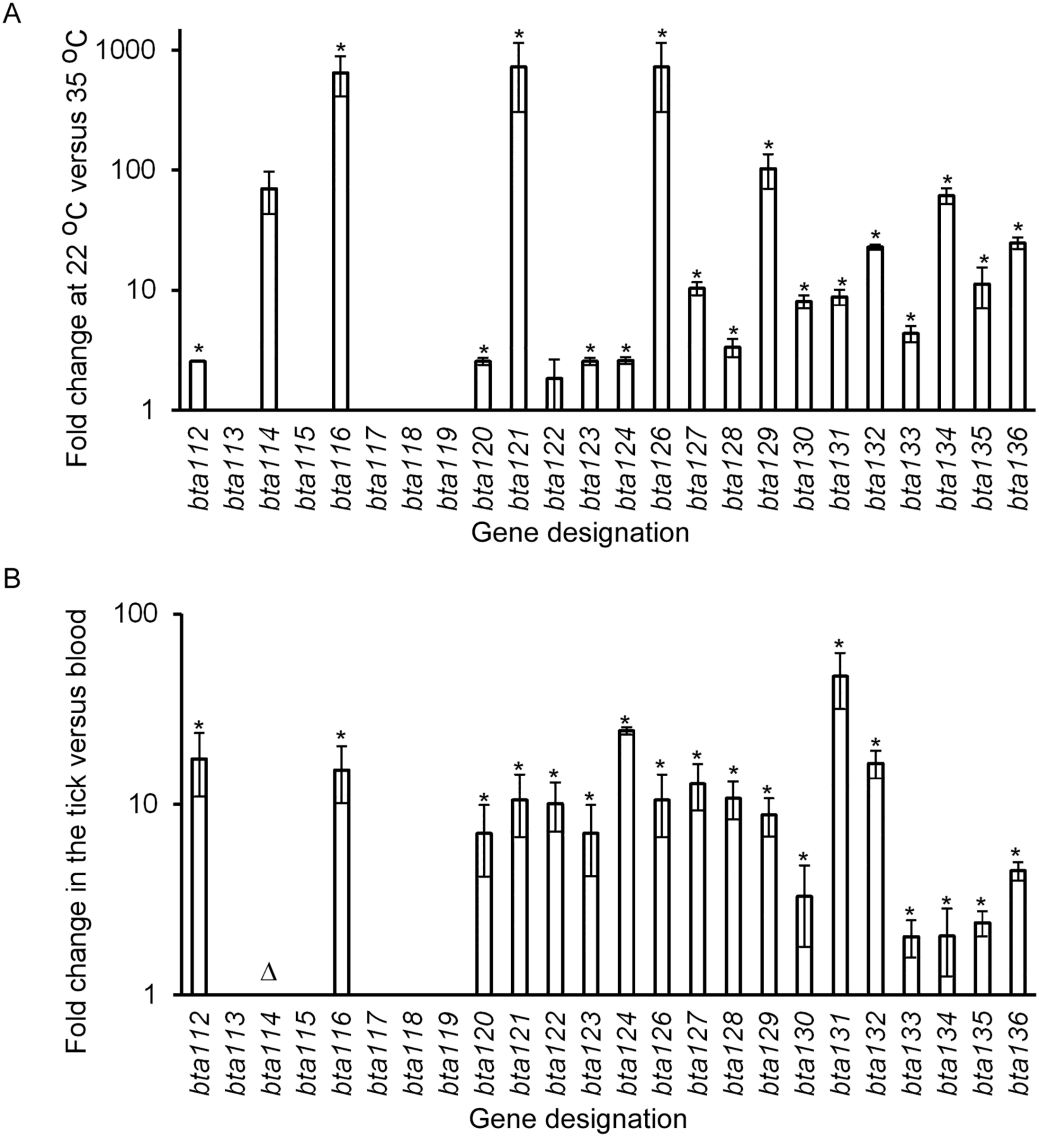
qRT-PCR evaluation of *bta* expression of 3’ end lp150 genes at 22°C and in the tick. The fold change of a given ORF is shown when *B*. *turicatae* was grown at 22°C relative to 35°C (A), and in infected ticks versus infected murine blood (B). Ninety-five percent confidence intervals are shown at the top of each bar, and asterisks indicate significant differences of expression between a given gene at 22°C versus 35°C (A) and infected ticks versus blood (B), as determined by a *t* test (*p*-value < 0.05). The triangle represents transcript that was only detected in infected ticks (B).

Increased gene expression ranged from 2.0- to > 500.0-fold when *B*. *turicatae* was cultivated at 22°C compared to spirochetes grown at 35°C (Fig 5A). Between infected unfed ticks and blood, expression was 2.0- to > 65.0-fold higher in the vector (Fig 5B). Numerical values for the fold change were unassigned for *bta113* to *bta115* and *bta117* to *bta119* because transcripts were undetectable for *bta113* and *bta115* in the tick and blood, suggesting lack of expression. For *bta114*, transcript was only detected in infected *O*. *turicata* and fold-change in expression could not be accurately calculated. Therefore, the gene was considered to be up-regulated in the tick. Similarly, *bta117* transcript was only detected in the tick, yet it remained unclear whether *bta117*, *bta118*, *bta119*, or all three ORFs were expressed. Of the ORFs that were validated *in vivo* and absent in the microarray GeneChips (*bta114*, *bta120*, *bta122*, *bta124*, and *bta127*), all were up-regulated by *B*. *turicatae* during tick colonization (Fig 5B). Also, a 10.0-fold increase in expression was detected for *bta121* in the tick relative to the blood, which microarrays initially indicated were not differentially regulated under the conditions tested (Fig 4 and S4 Table).

Of the three ORFs (*bta048*, *bta098*, and *bta110a*) that were identified by microarrays to be up-regulated by *B*. *turicatae* in murine blood compared to spirochetes cultivated at 22°C, primer and probe sets for qRT-PCR were designed to *bta048* and *bta098* (S5 Table). Optimal primers and probe specific to *bta110a* were not identified. Evaluating expression of *bta048* and *bta098* in the mammal and tick indicated that *bta048* and *bta098* were up-regulated over 0.62 (0.71–0.53 95% confidence interval) and 16.9-fold (17.72–16.01 95% confidence interval) during infection in the blood, respectively. Collectively, the microarray and qRT-PCR results suggest that genes on lp150 may have important roles in vector colonization and/or preparing the spirochetes for entry into the mammal.

## Discussion

With a focus on expression of ORFs localized to lp150, this study utilized a genomic and transcriptomic approach to begin identifying molecular events occurring during the life cycle of RF spirochetes. The linear megaplasmid is the largest in the genome of relapsing fever spirochetes, yet only 5–10% of ORFs have homologues outside of *Borrelia* [23] and a limited understanding of their transcriptional profiles exist. Moreover, incomplete genome assemblies [22, 23, 39, 40] have contributed to the gap in knowledge for the role of the megaplasmids in relapsing fever spirochete pathogenesis.

An analysis of ORFs on the identified 3’ end of lp150 supports previous findings that the ends of *Borrelia* plasmids are highly variable and may have been horizontally acquired [23, 41]. We detected a higher GC content of the ORFs toward the end of the plasmid, and identified direct and inverted repeats. Also, assessment of amino acid sequences from genes encoded on the 3’ end of lp150 indicates that while homologous genes appear to be unique to relapsing fever spirochetes, the paralogous sequences of each family are highly variable within species. With a given species of tick-borne relapsing fever spirochete having evolved to colonize a specific species of *Ornithodoros*, and in many instances to be vertically transmitted in the vector [42, 43], diversification of plasmid genes may have enabled the bacteria to adapt to unique environmental niches encountered within a species of tick.

To identify transcriptional changes *B*. *turicatae* undergoes in varying environmental conditions, *in vitro* and *in vivo* analyses were utilized. The ability of relapsing fever spirochetes to achieve densities over 1 x 10^7^ bacteria per ml of blood [44] enabled the purification of sufficient amounts of bacterial RNA from the mammal. As a substitute for conditions in the tick, from which the yield of recoverable spirochete RNA was very low (data not shown), we shifted the growth temperature of *B*. *turicatae* to 22°C, to mimic the vector’s environment for microarrays. Interestingly, a substantial portion of lp150 contained consecutive ORFs that were up-regulated at 22°C compared to spirochetes isolated from infected blood. Verification of genes on the 3’ end of lp150 in the tick supported the microarray findings, and determining whether genes on the plasmid are mono or polycistronically transcribed will be important toward revealing the pathogen’s regulatory mechanisms.

Evidence suggests the linear megaplasmids of relapsing fever spirochetes share ancestry to the 54 kb linear plasmid (lp54) of *B*. *burgdorferi* as they possess over 22 kb of orthologous sequence [23], yet the pathogens may have evolved differing regulatory mechanisms. Our findings and transcriptional studies in Lyme disease causing spirochetes [45–47] identified few orthologues (*bba64*, *bba68*, *bba04*, and bba09) that were similarly up-regulated by *B*. *turicatae* and *B*. *burgdorferi* in the pathogens respective tick vector or at 22°C. Moreover, in the mammal and at 35°C expression patterns of most lp54 orthologues also differed between *Borrelia* species. For example, expression of *B*. *burgdorferi bba31*, the orthologue of *bta098*, was not detected *in vitro* or *in vivo* [45, 46], while *bta098* was expressed 16.9-fold higher in mammalian blood compared to infected ticks. While the orthologues may function similarly their regulation differs between the two species of *Borrelia*. In the mammal, relapsing fever spirochetes replicate to over 1 x 10^7^ spirochetes per ml [44], attaining significantly higher densities compared to *B*. *burgdorferi* [48, 49]. BLAST analysis of *bta098* identified a bacteriophage terminase domain, and increased expression of the gene may be associated with replication and DNA packaging as the pathogens attain high densities in the blood.

Relapsing fever spirochetes cause a prolonged and cyclic mammalian infection that results from antigenic variation of variable major proteins (Vmps) [16, 50–52], which are up-regulated within three to five days after transmission [53]. However, once the pathogens enter the tick the molecular events utilized by the bacteria for vector adaptation are vague. Studies in *B*. *hermsii* and *B*. *turicatae* indicate that two populations of bacteria colonize an infected unfed tick, spirochetes in the midgut and salivary glands [6, 32]. As *B*. *hermsii* begins to transit from the midgut and colonizes the salivary glands Vmp-producing spirochetes are undetectable [53]. Transcriptional assessment of ORFs on lp150 suggests that a role of encoded surface proteins may be to facilitate midgut colonization, including evasion of tick immunity, environmental sensing, and adhesion and dissemination from the tissues. The procyclin domain of Bta121 may provide a clue toward the protein’s function in the tick, as indicated by the increased expression of *Trypanosoma brucei* procyclin upon entry into tsetse fly midgut [54, 55]. Two forms of procyclins occur, those with Glu-Pro and Gly-Pro-Glu-Glu-Thr amino acid repeats, and inactivation of the genes resulted in lower parasite loads in the insect midgut [54]. Given that our transcriptional analysis in the vector was performed using RNA from unfed infected *O*. *turicata*, future studies will assess temporal expression of *bta121* and additional genes that code for surface proteins during the spirochetes life cycle in the vector.

Subsets of ORFs within lp150 may also support salivary gland colonization and/or preadapt relapsing fever spirochetes as they enter the vertebrate host. Currently, three genes have been identified that are upregulated by relapsing fever spirochetes within the salivary glands, *B*. *hermsii* arthropod-associated outer membrane protein (Alp), the variable tick protein (Vtp), and the *Borrelia* repeat protein A (BrpA) [28, 32, 56, 57]. Alp and BrpA are encoded on the megaplasmid while the Vtp gene is localized on a 53 kb linear plasmid. The necessity of *alp* in the tick-mammalian infectious cycle remains unknown. For *brpA*, subsets of *B*. *turicatae* express the gene in the salivary glands yet it is dispensable for tick colonization and transmission [32]. Interestingly, Vtp is not required for spirochetes to colonize the tick but the gene is essential for establishing mammalian infection, as *vtp*-minus mutants of *B*. *hermsii* infected *O*. *hermsi* yet were not infectious to mice by tick bite [16].

Relapsing fever spirochetes circumvent innate immunity including complement, macrophage, and dendritic cell activation [58–60]. With transmission occurring within seconds of tick bite [14, 15], lp150 genes that are expressed in the salivary glands likely have roles in initiating early infection. With a rudimentary understanding of the molecular events occurring during vector colonization and early mammalian infection of relapsing fever spirochetes, candidates have been identified for further analysis. The developed genetic systems for targeted mutagenesis in relapsing fever spirochetes [16, 32, 33, 61, 62] will begin to identify and characterize essential mechanisms occurring in the tick and required for transmission.

## Supporting Information

S1 Fig Sequence reads generated from PacBio SMRT technology. Shown are the sequence length (x-axis), the number of sequences (left vertical axis), and the number sequences of length greater than a given sequence length (right vertical axis).
(TIF)

S2 Fig Venn diagrams of increased lp150 ORF expression when *B*. *turicatae* was grown under different *in vitro* and *in vivo* conditions. At 35°C, a majority of ORFs (65.2%) that were present on microarrays were similarly expressed compared to spirochetes isolated from infected blood (A). An increase of expression for 66.9% of ORFs was detected when spirochetes were cultivated at 22°C compared to 35°C (B).
(TIF)

S3 Fig qRT-PCR for *flaB* indicating similar transcript levels when spirochetes were grown at 22°C (blue squares) and 35°C (red diamonds). (TIF)

S4 Fig qPCR using DNA template to indicate similar genomic equivalents between *bta* genes of interest and *flaB*. *bta127*, *bta132*, and *bta135* are representative of the remaining ORFs that were evaluated *in vivo* by qRT-PCR. (TIF)

S1 Table ORF designation and locus. (DOCX)

S2 Table Amino acid identity between proteins encoded at the 3' end of lp150. (DOCX)

S3 Table Identified direct and inverted repeats at the 3’ end of *B*. *turicatae* lp150. (DOCX)

S4 Table Expression values from the microarray analysis. (DOCX)

S5 Table Primer and probe sets for ORFs on the 3' end of lp150. (DOCX)
