# Supplementary figures and images for: Transcriptional Profiling the 150 kb Linear Megaplasmid of Borrelia turicatae Suggests a Role in Vector Colonization and Initiating Mammalian Infection
Source: PLoS One. 2016 Feb 4;11(2):e0147707. doi: 10.1371/journal.pone.0147707 (PMC4741519; doi:10.1371/journal.pone.0147707)

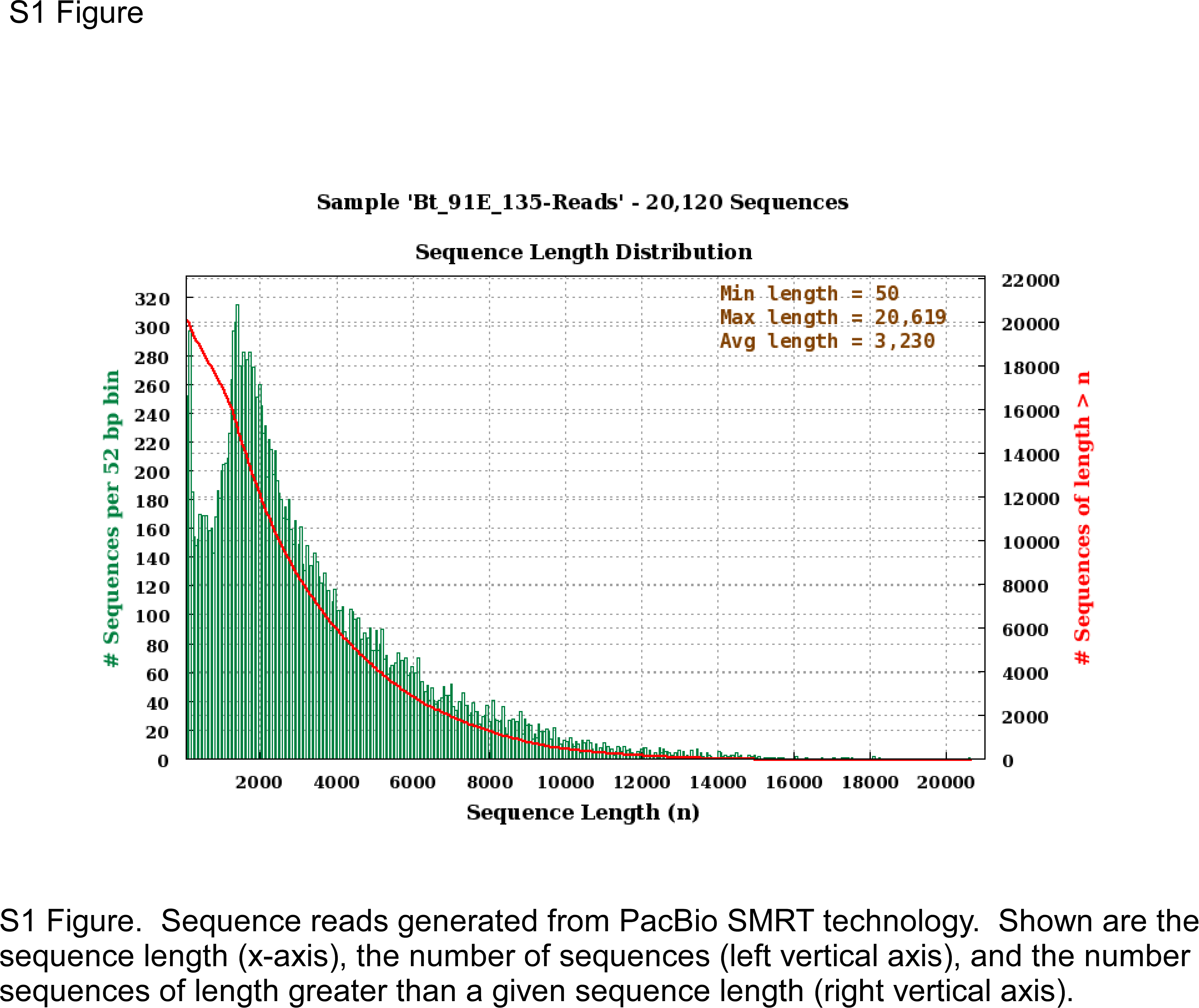

Supplement: S1 Fig — Shown are the sequence length (x-axis), the number of sequences (left vertical axis), and the number sequences of length greater than a given sequence length (right vertical axis). (TIF) [file pone.0147707.s001.tif]

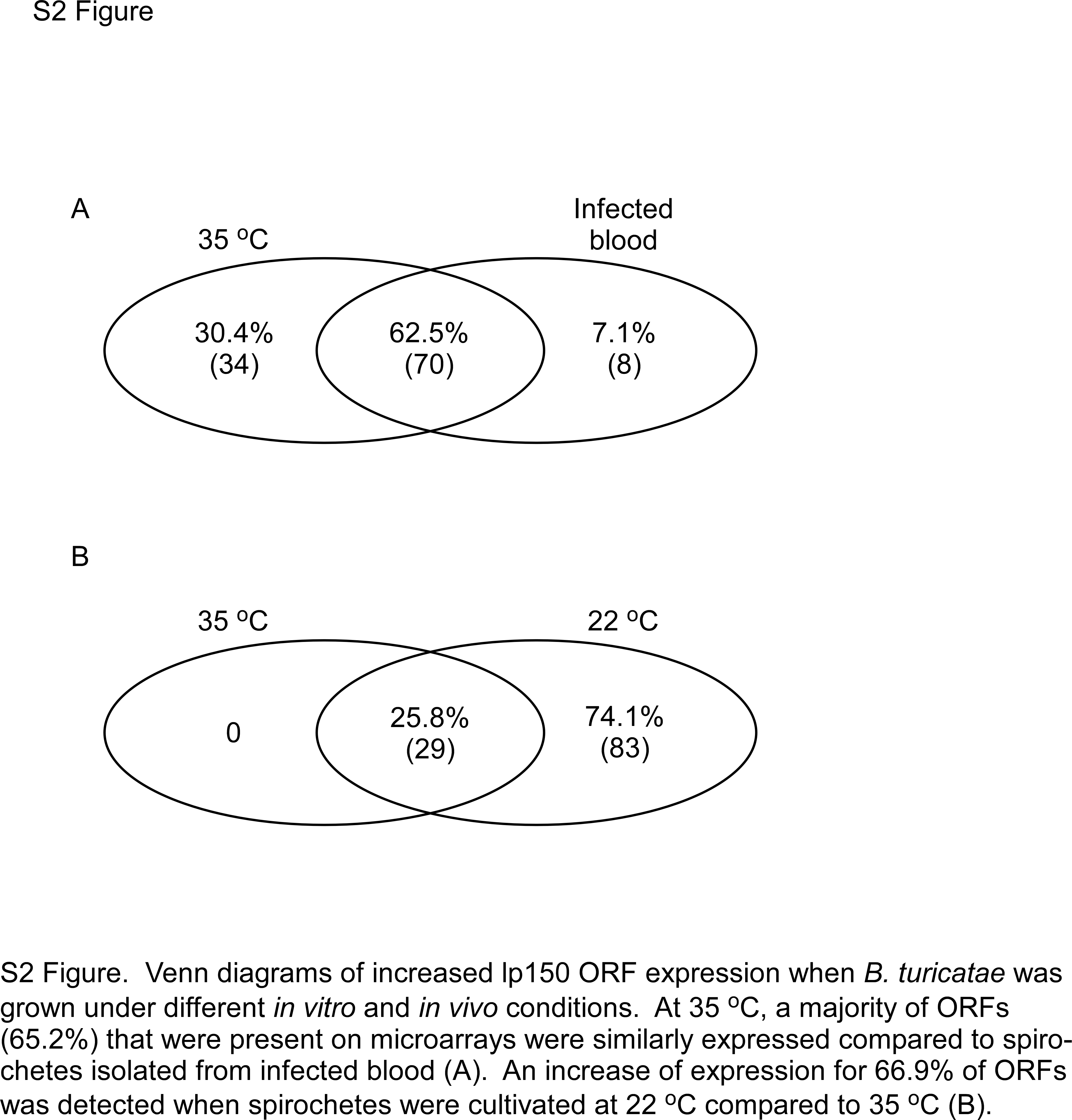

Supplement: S2 Fig — At 35°C, a majority of ORFs (65.2%) that were present on microarrays were similarly expressed compared to spirochetes isolated from infected blood (A). An increase of expression for 66.9% of ORFs was detected when spirochetes were cultivated at 22°C compared to 35°C (B). (TIF) [file pone.0147707.s002.tif]

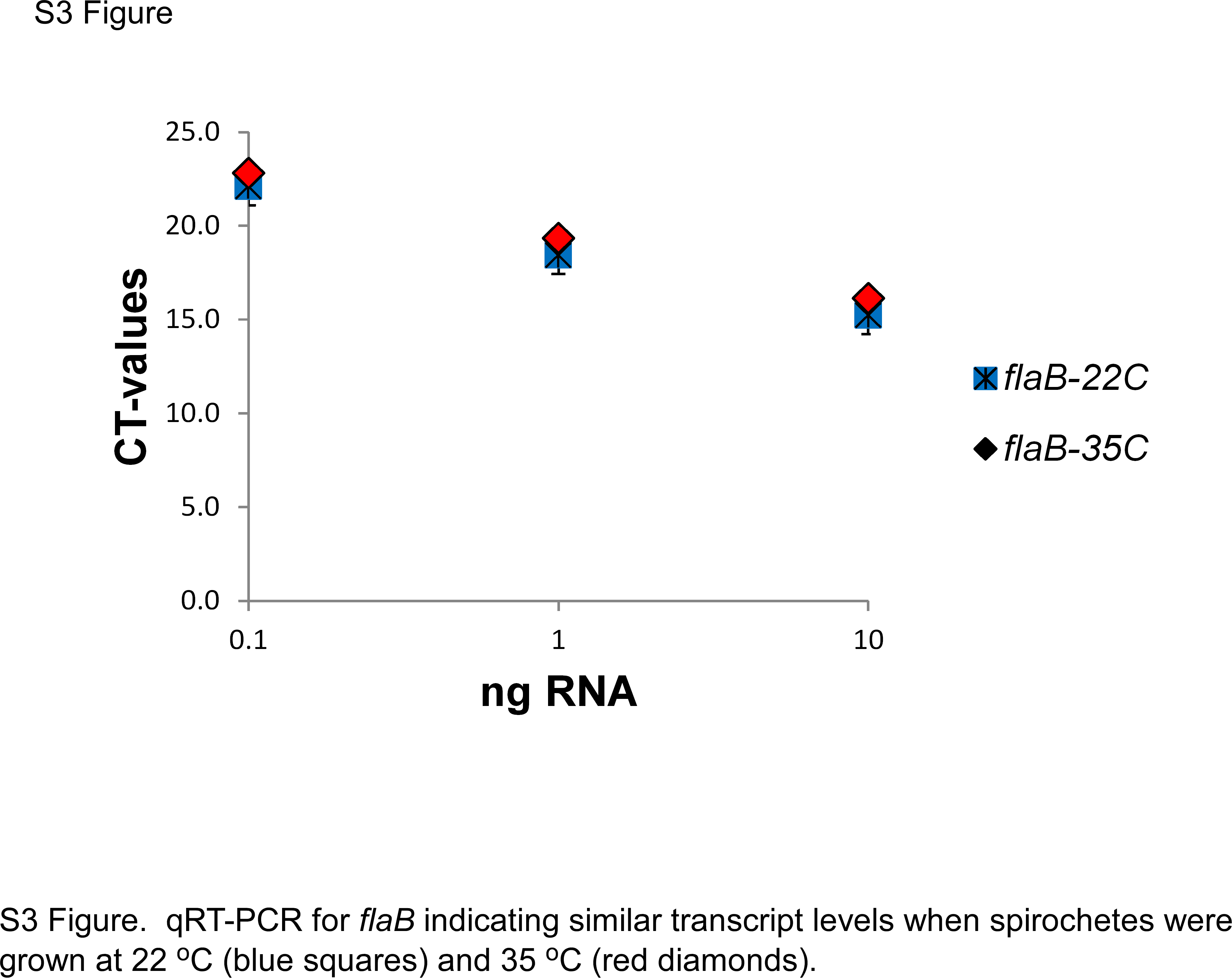

Supplement: S3 Fig — (TIF) [file pone.0147707.s003.tif]

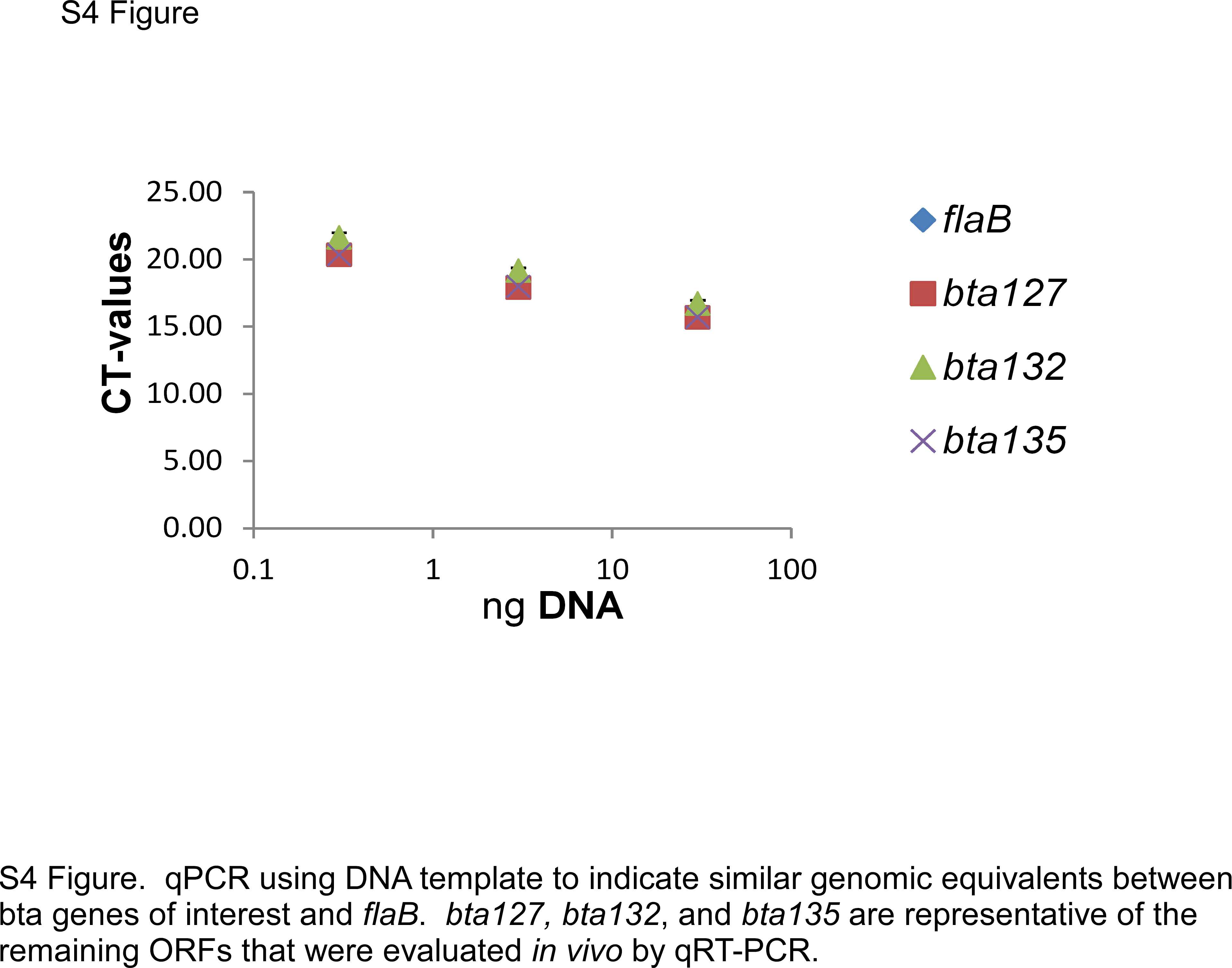

Supplement: S4 Fig — (TIF) [file pone.0147707.s004.tif]
