# Supplementary material for: Transcriptional Profiling the 150 kb Linear Megaplasmid of Borrelia turicatae Suggests a Role in Vector Colonization and Initiating Mammalian Infection
Source: PLoS One. 2016 Feb 4;11(2):e0147707. doi: 10.1371/journal.pone.0147707 (PMC4741519; doi:10.1371/journal.pone.0147707)
Supplement: S1 Table — (DOCX) [file pone.0147707.s005.docx]

| S1 Table. ORF designation and locus | |
| --- | --- |
| ORF | Locus |
| *bta*001 | 164_1300 |
| *bta*002 | 1435_2004 |
| *bta*003 | 3127_2051 |
| *bta*004 | 3452_3291 |
| *bta*004a | 4063_3644 |
| *bta*004b | 4438_4217 |
| *bta*004c | 5103_4987 |
| *bta*004d | 5221_5114 |
| *bta*006 | 5839_6621 |
| *bta*007 | 7290_8150 |
| *bta*008 | 8315_9100 |
| *bta*009 | 9267_10034 |
| *bta*010 | 10521_11333 |
| *bta*011 | 11598_11885 |
| *bta*012 | 11997_13046 |
| *bta*013 | 13283_14356 |
| *bta*014 | 14458_15360 |
| *bta*015 | 15584_16435 |
| *bta*015a | 16824_17714 |
| *bta*016 | 18386_19213 |
| *bta*016a | 19418_20230 |
| *bta*016b | 20473_21279 |
| *bta*017 | 21523_22491 |
| *bta*017a | 22970_22863 |
| *bta*018 | 24210_23143 |
| *bta*019 | 24661_25650 |
| *bta*020 | 27463_26483 |
| *bta*021 | 28722_27889 |
| *bta*022 | 29904_29017 |
| *bta*024 | 30943_30104 |
| *bta*025 | 32119_31274 |
| *bta*026 | 33242_32298 |
| *bta*027 | 34284_33493 |
| *bta*028 | 35106_34327 |
| *bta*029 | 36018_35149 |
| *bta*030 | 37234_36302 |
| *bta*031 | 38512_37469 |
| *bta*032 | 39852_38767 |
| *bta*033 | 40993_40247 |
| *bta*034 | 42170_41361 |
| *bta*035 | 43067_42228 |
| *bta*036 | 43960_43283 |
| *bta*037 | 44691_44071 |
| *bta*038 | 45503_44898 |
| *bta*039 | 46498_45752 |
| *bta*040 | 47556_46747 |
| *bta*041 | 48420_47629 |
| *bta*042 | 49448_48570 |
| *bta*043 | 50949_50068 |
| *bta*044 | 52026_51082 |
| *bta*046 | 52800_52651 |
| *bta*047 | 53608_52889 |
| *bta*048 | 54885_53923 |
| *bta*049 | 57001_54929 |
| *bta*050 | 57368_56982 |
| *bta*051 | 58193_57378 |
| *bta*052 | 58583_59401 |
| *bta*053 | 59681_60481 |
| *bta*054 | 60758_61636 |
| *bta*055 | 62393_61806 |
| *bta*056 | 63244_62630 |
| *bta*057 | 64184_63261 |
| *bta*058 | 65342_64245 |
| *bta*059 | 65936_65379 |
| *bta*060 | 67294_65972 |
| *bta*061 | 67513_67304 |
| *bta*062 | 67983_67567 |
| *bta*063 | 68405_67983 |
| *bta*064 | 69481_68408 |
| *bta*065 | 70090_69554 |
| *bta*066 | 70505_70116 |
| *bta*067 | 70876_70478 |
| *bta*068 | 71796_70870 |
| *bta*069 | 72458_71859 |
| *bta*070 | 73130_72534 |
| *bta*071 | 74359_73157 |
| *bta*072 | 75052_74786 |
| *bta*073 | 75628_75104 |
| *bta*074 | 76376_75750 |
| *bta*075 | 77050_76421 |
| *bta*076 | 78210_77101 |
| *bta*077 | 78806_78288 |
| *bta*078 | 79534_78890 |
| *bta*079 | 80098_79550 |
| *bta*080 | 80859_80191 |
| *bta*081 | 81469_80927 |
| *bta*082 | 82222_81671 |
| *bta*082a | 82961_82815 |
| *bta*085 | 83150_82989 |
| *bta*085a | 83281_83150 |
| *bta*086 | 83927_83325 |
| *bta*087 | 84535_83957 |
| *bta*088 | 85942_84749 |
| *bta*089 | 87892_86204 |
| *bta*090 | 89021_88074 |
| *bta*091 | 90007_89363 |
| *bta*092 | 90870_90214 |
| *bta*093 | 91471_90923 |
| *bta*094 | 91775_92575 |
| *bta*096 | 93543_93992 |
| *bta*096a | 94346_94230 |
| *bta*097 | 95305_94346 |
| *bta*098 | 97094_95739 |
| *bta*099 | 97648_97094 |
| *bta*099a | 98353_98216 |
| *bta*100 | 99183_98611 |
| *bta*101 | 99963_99214 |
| *bta*102 | 100505_99942 |
| *bta*103 | 101644_100538 |
| *bta*104 | 102508_102134 |
| *bta*105 | 102900_102508 |
| *bta*106 | 103098_102928 |
| *bta*107 | 104310_103207 |
| *bta*108 | 104956_104360 |
| *bta*109 | 105803_104946 |
| *bta*110 | 106129_105803 |
| *bta*110a | 106864_106727 |
| *bta*110b | 107306_107184 |
| *bta*111 | 108402_107554 |
| *bta*112 | 108858_110318 |
| *bta*113 | 110653_112125 |
| *bta*113a | 112426_112515 |
| *bta*114 | 112445_113494 |
| *bta*114a | 113707_113820 |
| *bta*115 | 113753_114937 |
| *bta*116 | 115227_116201 |
| *bta*117 | 116456_117763 |
| *bta*118 | 118015_119211 |
| *bta*119 | 119463_120599 |
| *bta*120 | 120842_122026 |
| *bta*121 | 122271_123605 |
| *bta*122 | 123873_124907 |
| *bta*123 | 125155_126444 |
| *bta*124 | 126719_127930 |
| *bta*125 | 128134_128253 |
| *bta*126 | 128186_129352 |
| *bta*127 | 129615_130676 |
| *bta*128 | 130922_132490 |
| *bta*129 | 132712_134175 |
| *bta*130 | 134577_135956 |
| *bta*131 | 136202_137803 |
| *bta*132 | 138032_139237 |
| *bta*133 | 140094_141557 |
| *bta*134 | 141788_142960 |
| *bta*135 | 143180_144595 |
| *bta*136 | 144810_146264 |
| *bta*137 | 146497_146586 |
| *bta*138 | 146733_147044 |
| *bta*139 | 147468_148523 |
|  |  |
