# Supplementary material for: Transcriptional Profiling the 150 kb Linear Megaplasmid of Borrelia turicatae Suggests a Role in Vector Colonization and Initiating Mammalian Infection
Source: PLoS One. 2016 Feb 4;11(2):e0147707. doi: 10.1371/journal.pone.0147707 (PMC4741519; doi:10.1371/journal.pone.0147707)
Supplement: S2 Table — (DOCX) [file pone.0147707.s006.docx]

| S2 Table. Amino acid identity between proteins encoded at the 3' end of lp150 | | | | | | | | | | | | | | | | | | | | | | | | |
| --- | --- | --- | --- | --- | --- | --- | --- | --- | --- | --- | --- | --- | --- | --- | --- | --- | --- | --- | --- | --- | --- | --- | --- | --- |
|  | **112** | **113** | **114** | **115** | **116** | **117** | **118** | **119** | **120** | **121** | **122** | **123** | **124** | **126** | **127** | **128** | **129** | **130** | **131** | **132** | **133** | **134** | **135** | **136** |
| **112** |  | 32.3 | 19.6 | 19.6 | 19.3 | 20.0 | 20.4 | 19.8 | 17.4 | 22.4 | 19.2 | 19.6 | 17.7 | 22.1 | 19.6 | 29.0 | 32.7 | 30.3 | 20.9 | 23.7 | 28.5 | 21.0 | 31.4 | 33.3 |
| **113** | 32.3 |  | 23.3 | 16.7 | 17.0 | 14.1 | 17.9 | 19.2 | 15.7 | 15.0 | 17.4 | 18.6 | 15.4 | 19.4 | 17.5 | 31.8 | 35.3 | 37.4 | 16.4 | 24.4 | 30.0 | 18.8 | 31.9 | 32.1 |
| **114** | 19.6 | 23.3 |  | 16.3 | 18.3 | 15.1 | 17.5 | 18.8 | 13.4 | 16.4 | 18.9 | 16.9 | 16.9 | 19.9 | 18.1 | 22.8 | 24.6 | 19.1 | 12.4 | 24.4 | 19.7 | 22.6 | 19.8 | 19.6 |
| **115** | 19.6 | 16.7 | 16.3 |  | 44.3 | 41.8 | 41.2 | 39.9 | 34.8 | 42.8 | 34.8 | 38.1 | 35.9 | 40.4 | 37.4 | 13.8 | 16.7 | 16.1 | 11.8 | 21.7 | 16.8 | 17.6 | 17.5 | 18.0 |
| **116^a^** | 19.3 | 17.0 | 18.3 | 44.3 |  | 45.8 | 50.1 | 53.8 | 49.5 | 42.9 | 57.1 | 48.6 | 47.9 | 49.7 | 55.1 | 17.4 | 19.3 | 15.9 | 11.8 | 22.3 | 14.3 | 21.2 | 17.9 | 17.6 |
| **117^a^** | 20.0 | 14.1 | 15.1 | 41.8 | 45.8 |  | 81.3 | 76.2 | 66.4 | 41.4 | 66.0 | 71.8 | 53.6 | 50.5 | 53.7 | 17.7 | 14.8 | 16.2 | 11.9 | 20.1 | 18.0 | 14.3 | 15.2 | 17.1 |
| **118^a^** | 20.4 | 17.9 | 17.5 | 41.2 | 50.1 | 81.3 |  | 88.2 | 67.1 | 39.7 | 69.3 | 86.3 | 55.0 | 52.8 | 58.1 | 19.1 | 19.4 | 15.0 | 13.4 | 19.9 | 17.0 | 20.4 | 18.3 | 18.8 |
| **119^a^** | 19.8 | 19.2 | 18.8 | 39.9 | 53.8 | 76.2 | 88.2 |  | 68.6 | 39.9 | 72.1 | 81.8 | 52.1 | 51.1 | 61.2 | 19.4 | 20.9 | 15.2 | 13.1 | 21.8 | 16.5 | 20.4 | 19.0 | 19.5 |
| **120^a^** | 17.4 | 15.7 | 13.4 | 34.8 | 49.5 | 66.4 | 67.1 | 68.6 |  | 33.9 | 67.1 | 73.2 | 47.8 | 45.3 | 55.4 | 18.5 | 19.0 | 16.6 | 11.1 | 18.2 | 15.8 | 18.7 | 18.6 | 17.4 |
| **121** | 22.4 | 15.0 | 16.4 | 42.8 | 42.9 | 41.4 | 39.7 | 39.9 | 33.9 |  | 38.5 | 40.3 | 38.3 | 42.7 | 42.4 | 18.0 | 15.3 | 14.8 | 14.5 | 20.6 | 17.2 | 14.1 | 17.2 | 16.0 |
| **122^a^** | 19.2 | 17.4 | 18.9 | 34.8 | 57.1 | 66.0 | 69.3 | 72.1 | 67.1 | 38.5 |  | 63.8 | 46.3 | 49.0 | 63.5 | 18.5 | 20.9 | 16.2 | 10.7 | 19.5 | 18.0 | 19.9 | 18.4 | 19.0 |
| **123^a^** | 19.6 | 18.6 | 16.9 | 38.1 | 48.6 | 71.8 | 86.3 | 81.8 | 73.2 | 40.3 | 63.8 |  | 55.6 | 47.9 | 55.9 | 19.8 | 20.6 | 12.2 | 12.4 | 20.9 | 17.0 | 19.2 | 18.7 | 18.8 |
| **124^a^** | 17.7 | 15.4 | 16.9 | 35.9 | 47.9 | 53.6 | 55.0 | 52.1 | 47.8 | 38.3 | 46.3 | 55.6 |  | 46.4 | 43.8 | 17.9 | 18.7 | 11.1 | 12.7 | 20.3 | 15.5 | 17.4 | 15.7 | 15.8 |
| **126^a^** | 22.1 | 19.4 | 19.9 | 40.4 | 49.7 | 50.5 | 52.8 | 51.1 | 45.3 | 42.7 | 49.0 | 47.9 | 46.4 |  | 51.9 | 21.8 | 18.7 | 15.4 | 12.3 | 23.9 | 18.3 | 14.5 | 16.8 | 17.1 |
| **127^a^** | 19.6 | 17.5 | 18.1 | 37.4 | 55.1 | 53.7 | 58.1 | 61.2 | 55.4 | 42.4 | 63.5 | 55.9 | 43.8 | 51.9 |  | 18.5 | 21.4 | 16.9 | 11.1 | 20.2 | 15.5 | 19.6 | 18.2 | 17.2 |
| **128** | 29.0 | 31.8 | 22.8 | 13.8 | 17.4 | 17.7 | 19.1 | 19.4 | 18.5 | 18.0 | 18.5 | 19.8 | 17.9 | 21.8 | 18.5 |  | 34.0 | 25.4 | 21.3 | 24.4 | 27.9 | 19.4 | 30.0 | 30.5 |
| **129** | 32.7 | 35.3 | 24.6 | 16.7 | 19.3 | 14.8 | 19.4 | 20.9 | 19.0 | 15.3 | 20.9 | 20.6 | 18.7 | 18.7 | 21.4 | 34.0 |  | 30.6 | 22.9 | 23.9 | 32.8 | 18.9 | 32.8 | 31.1 |
| **130** | 30.3 | 37.4 | 19.1 | 16.1 | 15.9 | 16.2 | 15.0 | 15.2 | 16.6 | 14.8 | 16.2 | 12.2 | 11.1 | 15.4 | 16.9 | 25.4 | 30.6 |  | 18.0 | 24.1 | 28.0 | 20.2 | 27.2 | 30.7 |
| **131** | 20.9 | 16.4 | 12.4 | 11.8 | 11.8 | 11.9 | 13.4 | 13.1 | 11.1 | 14.5 | 10.7 | 12.4 | 12.7 | 12.3 | 11.1 | 21.3 | 22.9 | 18.0 |  | 20.3 | 18.9 | 14.7 | 18.1 | 18.9 |
| **132** | 23.7 | 24.4 | 24.4 | 21.7 | 22.3 | 20.1 | 19.9 | 21.8 | 18.2 | 20.6 | 19.5 | 20.9 | 20.3 | 23.9 | 20.2 | 24.4 | 23.9 | 24.1 | 20.3 |  | 27.5 | 23.8 | 23.5 | 25.9 |
| **133** | 28.5 | 30.0 | 19.7 | 16.8 | 14.3 | 18.0 | 17.0 | 16.5 | 15.8 | 17.2 | 18.0 | 17.0 | 15.5 | 18.3 | 15.5 | 27.9 | 32.8 | 28.0 | 18.9 | 27.5 |  | 21.1 | 30.6 | 30.0 |
| **134** | 21.0 | 18.8 | 22.6 | 17.6 | 21.2 | 14.3 | 20.4 | 20.4 | 18.7 | 14.1 | 19.9 | 19.2 | 17.4 | 14.5 | 19.6 | 19.4 | 18.9 | 20.2 | 14.7 | 23.8 | 21.1 |  | 18.8 | 19.6 |
| **135** | 31.4 | 31.9 | 19.8 | 17.5 | 17.9 | 15.2 | 18.3 | 19.0 | 18.6 | 17.2 | 18.4 | 18.7 | 15.7 | 16.8 | 18.2 | 30.0 | 32.8 | 27.2 | 18.1 | 23.5 | 30.6 | 18.8 |  | 37.1 |
| **136** | 33.3 | 32.1 | 19.6 | 18.0 | 17.6 | 17.1 | 18.8 | 19.5 | 17.4 | 16.0 | 19.0 | 18.8 | 15.8 | 17.1 | 17.2 | 30.5 | 31.1 | 30.7 | 18.9 | 25.9 | 30.0 | 19.6 | 37.1 |  |

^a^ Green and yellow shaded boxes represent amino acid identity ranging between 49.5 to 59% and 60 to 85%, respectively.
