# Supplementary material for: Transcriptional Profiling the 150 kb Linear Megaplasmid of Borrelia turicatae Suggests a Role in Vector Colonization and Initiating Mammalian Infection
Source: PLoS One. 2016 Feb 4;11(2):e0147707. doi: 10.1371/journal.pone.0147707 (PMC4741519; doi:10.1371/journal.pone.0147707)
Supplement: S3 Table — (DOCX) [file pone.0147707.s007.docx]

| S3 Table. Identified direct and inverted repeats at the 3’ end of *B. turicatae* lp150. | | | | | |
| --- | --- | --- | --- | --- | --- |
| Repeat ORF location | Length | Direct Repeat (DR) | DR length | Inverted Repeat (IR) | IR length |
| *bta116* | 1256 | TTTA | 4 | AGCCTTAAACGTACACGTTTAAGGCTTTT | 29 |
| *bta117* | 1579 | TTAA | 4 | ACGTGTATATTTAAGGCTTTT | 21 |
| *bta118* | 1459 | TATA | 4 | TTATTATAATAATATAATATA | 21 |
| *bta119-120* | 2835 | AAT | 3 | TTGTATATATATAAATTATGT | 21 |
| *bta120* | 1447 | AAT | 3 | TTGTATATATATAAATTATGT | 21 |
| *bta121-123* | 4431 | TAT | 3 | ATATATAGAAAATATATAAATTAT | 24 |
| *bta122-123* | 2852 | TTTA | 4 | AGCCTTAAACGTATAGATTTAAA | 23 |
| *bta123* | 1562 | TTTA | 4 | AGCTTTAAACGTATAAGTTTAAGA | 24 |
| *bta124-126* | 2936 | TTAA | 4 | ACATGTACGTTTAAGGCTTTT | 21 |
| *bta125-127* | 2774 | TTAA | 4 | ACGTGTACGTTTAAGGCTTTT | 21 |
| *bta128* | 1850 | TTTA | 4 | AGCCTTAAATGTACACGTTTAAGGCTTT | 28 |
| *bta131-132* | 3420 | AAT | 3 | AATAAGTTTATATAAAAAAAA | 21 |
| *bta134-138* | 5737 | TTA | 3 | TTATATTATGCTAACATAATAA | 22 |
| *bta135-136* | 1661 | GTAATAA | 7 | TACTAATAATAATATTATTGT | 21 |
| *bta139* | 1265 | GGT | 3 | TAAGCCTTAGATGTTAATCTAAGGT | 25 |
