# Supplementary material for: Transcriptional Profiling the 150 kb Linear Megaplasmid of Borrelia turicatae Suggests a Role in Vector Colonization and Initiating Mammalian Infection
Source: PLoS One. 2016 Feb 4;11(2):e0147707. doi: 10.1371/journal.pone.0147707 (PMC4741519; doi:10.1371/journal.pone.0147707)
Supplement: S4 Table — (DOCX) [file pone.0147707.s008.docx]

| S4 Table. Expression values from the microarray analysis | | | |
| --- | --- | --- | --- |
| ORF | Fold change at 22 ^o^C relative to blood | Fold change 22 ^o^C relative to 35 ^o^C | Fold change at 35 ^o^C relative to blood |
| *bta001* | UD ^A^ | UD | UD |
| *bta002* | UD | UD | UD |
| *bta003* | UD | UD | UD |
| *bta004* | UD | UD | UD |
| *bta004a* | 18.05 | 7.71 | - ^B^ |
| *bta004b* | 4.53 | 9.41 | -2.08 |
| *bta004c* | UD | UD | UD |
| *bta004d* | UD | UD | UD |
| *bta006* | 9.16 | 1.62 | 5.65 |
| *bta007* | 2.43 | 8.47 | - |
| *bta008* | 16.43 | 3.47 | 4.73 |
| *bta009* | - | 1.97 | - |
| *bta010* | 106.26 | 31.94 | 3.3 |
| *bta011* | -1.47 | 1.54 | 2.12 |
| *bta012* | 5.51 | 5.74 | -1.04 |
| *bta013* | 15.27 | 5.71 | 2.68 |
| *bta014* | 4.30 | 2.83 | 1.52 |
| *bta015* | 44.84 | 45.54 | -1.02 |
| *bta015a* | 5.96 | 43.09 | - |
| *bta016* | - | - | - |
| *bta016a* | - | - | - |
| *bta016b* | 4.65 | 4.70 | - |
| *bta017* | UD | UD | UD |
| *bta017a* | UD | UD | UD |
| *bta018* | 34.41 | 9.32 | 3.73 |
| *bta019* | UD | UD | UD |
| *bta020* | UD | UD | UD |
| *bta021* | UD | UD | UD |
| *bta022* | 5.07 | - | 3.77 |
| *bta024* | 8.15 | 3.29 | 2.48 |
| *bta025* | 23.39 | 4.32 | 5.41 |
| *bta026* | 12.92 | 3.92 | 3.29 |
| *bta027* | 5.75 | 2.44 | 2.36 |
| *bta028* | 14.24 | 5.15 | - |
| *bta029* | 3.38 | 4.36 | -1.29 |
| *bta030* | UD | UD | UD |
| *bta031* | 13.35 | 1.57 | 8.53 |
| *bta032* | 11.64 | 5.19 | 2.24 |
| *bta033* | 35.73 | 3.69 | 9.67 |
| *bta034* | 4.40 | 3.64 | 1.21 |
| *bta035* | 3.51 | 2.66 | 1.32 |
| *bta036* | 12.10 | 6.61 | 1.83 |
| *bta037* | 6.60 | 4.59 | 1.06 |
| *bta038* | 3.30 | 3.88 | - |
| *bta039* | 2.90 | 2.12 | 1.36 |
| *bta040* | 6.66 | 5.00 | 1.33 |
| *bta041* | 45.52 | 4.71 | 9.66 |
| *bta042* | 4.47 | 3.46 | 1.29 |
| *bta043* | 1.07 | 7.79 | - |
| *bta044* | 1.23 | 2.99 | - |
| *bta046* | UD | UD | UD |
| *bta047* | 22.92 | 9.97 | 2.3 |
| *bta048* | -18.83 | - | -107.44 |
| *bta049* | - | - | - |
| *bta050* | - | - | -6.2 |
| *bta051* | - | 3.03 | 1.18 |
| *bta052* | 1.84 | - | 2.43 |
| *bta053* | 4.24 | 3.32 | 1.28 |
| *bta054* | 2.10 | 8.17 | - |
| *bta055* | 5.19 | 1.87 | 2.77 |
| *bta056* | 1.59 | 5.64 | - |
| *bta057* | 4.64 | 3.94 | 1.18 |
| *bta058* | 35.58 | 7.19 | 4.94 |
| *bta059* | -1.13 | 1.57 | - |
| *bta060* | 3.35 | 2.60 | 1.29 |
| *bta061* | - | -1.82 | - |
| *bta062* | 0.02 | - | - |
| *bta063* | -1.98 | - | - |
| *bta064* | -1.20 | - | - |
| *bta065* | -1.02 | - | - |
| *bta066* | 2.39 | 4.54 | - |
| *bta067* | 3.76 | 7.05 | - |
| *bta068* | - | - | - |
| *bta069* | 2.73 | 8.97 | -3.28 |
| *bta070* | 1.78 | 3.08 | - |
| *bta071* | 2.31 | 2.38 | -1.03 |
| *bta072* | 2.67 | 4.34 | - |
| *bta073* | 2.53 | 6.27 | -2.48 |
| *bta074* | 6.87 | 5.70 | 1.94 |
| *bta075* | 3.34 | 6.67 | - |
| *bta076* | 15.27 | 6.51 | 2.34 |
| *bta077* | 10.07 | 6.34 | - |
| *bta078* | 1.54 | 5.23 | - |
| *bta079* | 3.01 | 4.30 | - |
| *bta080* | 7.12 | 4.08 | 1.75 |
| *bta081* | 29.22 | 7.01 | 4.17 |
| *bta082* | 3.06 | 6.09 | -1.19 |
| *bta082a* | UD | UD | UD |
| *bta085* | UD | UD | UD |
| *bta085a* | UD | UD | UD |
| *bta086* | 3.63 | 3.16 | 1.15 |
| *bta087* | 19.29 | 1.43 | 13.47 |
| *bta088* | 1.89 | -1.80 | 3.4 |
| *bta089* | 2.60 | - | 5.58 |
| *bta090* | 2.57 | 1.17 | 2.2 |
| *bta091* | UD | UD | UD |
| *bta092* | UD | UD | UD |
| *bta093* | 1.91 | - | 2.93 |
| *bta094* | 1.73 | 7.93 | - |
| *bta096* | UD | UD | UD |
| *bta096a* | UD | UD | UD |
| *bta097* | 1.35 | - | 1.61 |
| *bta098* | -6.14 | - | -3.51 |
| *bta099* | -1.23 | - | - |
| *bta099a* | UD | UD | UD |
| *bta100* | 2.36 | 3.47 | - |
| *bta101* | -1.83 | - | - |
| *bta102* | 2.45 | 1.43 | 3.1 |
| *bta103* | 1.00 | 2.07 | - |
| *bta104* | -1.27 | 2.04 | - |
| *bta105* | -1.02 | - | - |
| *bta106* | 0.42 | - | - |
| *bta107* | -1.57 | 5.70 | - |
| *bta108* | -1.49 | - | - |
| *bta109* | -1.32 | 1.57 | - |
| *bta110* | - | - | - |
| *bta110a* | -78.60 | - | -39.48 |
| *bta110b* | UD | UD | UD |
| *bta111* | UD | UD | UD |
| *bta112* | 16.48 | 1.96 | 8.4 |
| *bta113* | 4.32 | 3.02 | - |
| *bta113a* | 31.35 | 16.88 | 1.86 |
| *bta114* | UD | UD | UD |
| *bta114a* | UD | UD | UD |
| *bta115* | 15.42 | 8.61 | - |
| *bta116* | 20.28 | 3.90 | - |
| *bta117* | 7.05 | 6.02 | - |
| *bta118* | 4.08 | 3.09 | 1.32 |
| *bta119* | 12.94 | 5.50 | 2.35 |
| *bta120* | UD | UD | UD |
| *bta121* | -1.58 | 4.04 | - |
| *bta122* | UD | UD | UD |
| *bta123* | 7.84 | 7.90 | -1.01 |
| *bta124* | UD | UD | UD |
| *bta125* | UD | UD | UD |
| *bta126* | 11.60 | 2.59 | 4.48 |
| *bta127* | UD | UD | UD |
| *bta128* | 3.66 | 2.64 | 1.39 |
| *bta129* | 33.66 | 5.67 | 5.94 |
| *bta130* | 5.75 | 3.81 | 1.51 |
| *bta131* | 30.93 | 3.4 | 9.1 |
| *bta132* | 41.15 | 5.62 | 7.32 |
| *bta133* | 6.55 | 2.22 | 2.95 |
| *bta134* | 9.88 | 8.4 | 1.18 |
| *bta135* | 30.91 | 5.33 | 5.8 |
| *bta136* | 3.17 | 13.98 | -4.42 |
| *bta137* | UD | UD | UD |
| *bta138* | -1.33 | 3.92 | - |
| *bta139* | UD | UD | UD |

^A^ UD: undetermined

^B^ (-): transcript was undetectable or below the threshold set for negative controls.
