# Supplementary material for: Transcriptional Profiling the 150 kb Linear Megaplasmid of Borrelia turicatae Suggests a Role in Vector Colonization and Initiating Mammalian Infection
Source: PLoS One. 2016 Feb 4;11(2):e0147707. doi: 10.1371/journal.pone.0147707 (PMC4741519; doi:10.1371/journal.pone.0147707)
Supplement: S5 Table — (DOCX) [file pone.0147707.s009.docx]

| S5 Table. Primer and probe sets for ORFs on the 3' end of lp150 | |
| --- | --- |
| *bta* designation | Sequence |
| 112 F | 5' - TTCTGATGAGGATAAAGGCGTA |
| 112 R | 5' - GTATTTATAGCCTCCAAAGCCA |
| 112 Probe | 5' - 6FAM-TTCCCGAAGGACGTTAGCCCTTAAA--BBQ |
| 113F | 5' - AGAAAATGAGGCTTATGCAGC |
| 113 R | 5' - CCTCTGATCTACCAATCTTCACATC |
| 113 Probe | 5' - 6FAM-TGTTATATGCTGTTTCTGTGGCACATCG--BBQ |
| 114 F | 5' - TGTGCTTGATCCTAAGAAGCC |
| 114 R | 5' - TCTGGTGCTTGTTAAAACGTC |
| 114 Probe | 5' - 6FAM-TGAGGCCTTGTTGGGTAGGATGGGT--BBQ |
| 115 F | 5' - TGGTTAAAAGTCCACGTGTGA |
| 115 R | 5' - CATCAGCTGCTTTCTTCTCAAT |
| 115 Probe | 5' - 6FAM-TGGATGGCTTGATGTTGATGAGCAGG--BBQ |
| 116 F | 5' - AAAGAGATGGTTAAAAATCCGC |
| 116 R | 5' - TGCATCATCAGAAACACCCT |
| 116 Probe | 5' - 6FAM-TGCCTGGCTTGATGATGCTGATCAG--BBQ |
| 117 F | 5' - TGAGATTAAAGAGGACGCCAAA |
| 117 R | 5' - CCAGCTTCGGTACTTTCTTCA |
| 117 Probe | 5' - 6FAM-AGCCAAAGAAGCTAAAGAAGCCGC--BBQ |
| 120 F | 5' - TGAGATTAAAGAGGACGCCAAA |
| 120 R | 5' - CCAGCTTCGGTACTTTCTTCA |
| 120 Probe | 5' - 6FAM-AGCCAAAGAAGCTAAAGAAGCCGC--BBQ |
| 121 F | 5' - GGCTGTTGTTTATTATGCACC |
| 121 R | 5' - ATTGAGCATGATGAAGCTTTG |
| 121 Probe | 5' - 6FAM-TGGCTTGATGCTGATGATCGTGCCA--BBQ |
| 122 F | 5' - AGGAGCTGATGTTGTGATTGA |
| 122 R | 5’ - GCCTGCATCTGTTGTACCT |
| 122 Probe | 5' - 6FAM-AGCTAGAGGTAAAGCTTTGAGAGTTATGCA--BBQ |
| 123 F | 5' - TGGAGGACAAAGTTATCGATCC |
| 123 R | 5' - ATGAACATCACCACTATCACGG |
| 123 Probe | 5' - 6FAM-ACCAATTGCCTTCCTTCCTGGTCCA--BBQ |
| 124 F | 5' - TAAAGCCACCGTTGCAGTT |
| 124 R | 5' - CGCACCACTTTCTTCAACAGT |
| 124 Probe | 5' - 6FAM-TCTCCAGGTTCGCCAGCTTCGG--BBQ |
| 126 F | 5' - AAAGCTATAGAGGGTGTTTCTGA |
| 126 R | 5' - CGCACCACTTGATTTGACA |
| 126 Probe | 5' - 6FAM-TCCTTGGGCAGCTTCGGCAG--BBQ |
| 127 F | 5' - GGTACAACAGATGAAGGCGTA |
| 127 R | 5' - AGCATTTGCAAATTTATCTTCG |
| 127 Probe | 5' - 6FAM-AGTGATCACGATCGGGATTCCATGCA--BBQ |
| 128 F | 5' - GGATTCTGGTGAAGTGTATCGTG |
| 128 R | 5' - GCATTTCTAACCCCTTCTTCTG |
| 128 Probe | 5' - 6FAM-TCCCCAACCTTCTCAGCACCTAACA--BBQ |
| 129 F | 5' - GGATGTATACACATGGCTGTCTG |
| 129 R | 5' - CTCGTAATTGCTCTCTGAAGTCA |
| 129 Probe | 5' - 6FAM-AGCTTCTAACCATGCCGCATCTTGG--BBQ |
| 130 F | 5' - GAATAAGGCTGAGGAGAGTCAGA |
| 130 R | 5' - GCAGCATATTTGCTCCCC |
| 130 Probe | 5' - 6FAM-TGAGATGCGATGCATAGCCACGACT--BBQ |
| 131 F | 5' - TGAAGTGAAAGACAGAGTTGCATA |
| 131 R | 5' - ACTTAGAAACACCAAAAGCTCC |
| 131 Probe | 5' - 6FAM-TGCAGGGCTAATTGGTCAAGCAAAGC--BBQ |
| 132 F | 5' - CCGGTTGAAGCATCTACAGT |
| 132 R | 5' - AGGGTATCCCAAATGTACCAAG |
| 132 Probe | 5' - 6FAM-TACCCCTACGCTCCCCATTTCAGAA--BBQ |
| 133 F | 5' - GATCCTAACCCTGACACGGT |
| 133 R | 5' - AAATGCAATTGCTTCAAGCTC |
| 133 Probe | 5' - 6FAM-AGGCCGACATCCACCCTGCA--BBQ |
| 134 F | 5' - GTTGGAACAATTAGGTGCTGAG |
| 134 R | 5' - GGATTAGAATCCTTAAAGGCCC |
| 134 Probe | 5' - 6FAM-TCGTACCAAGCAAGCTCCCCCTCC--BBQ |
| 135 F | 5' - TGTTAGGTGAGTTAGGCGCTAC |
| 135 R | 5' - AGTGTTGTTTTTCATCATCCCC |
| 135 Probe | 5' - 6FAM-TCGCGAACCTCGTCCTGGGTC--BBQ |
| 136 F | 5' - GCTGAGCTTGAAGATGAGGCT |
| 136 R | 5' - ACGTTTTATGACACAGAGGACC |
| 136 Probe | 5' - 6FAM-AGTGGGCGTGGACGTATATGCAGGA--BBQ |
| 048 F | 5’ - ATCCACTCTATCTTGCAGGTCA |
| 048 R | 5’ - TTGTGCCAATAGACCAACG |
| 048 Probe | 5’ - 6FAM-ACTCCATGAACAGATTC+ATC+ACGC--BBQ^A^ |
| 098 F | 5’ - GCTTACAAGGCCCGAGTAC |
| 098 R | 5’ - AGCAGTATTGTCCATACCAACAG |
| 098 Probe | 5’ - 6FAM-TGGACTGCTAGTGCTGATGCGTGT--BBQ |

^A^: (+) represent locations of locked nucleic acids to increase the melting temperature of the probe
